# Alternative Randomized Trial Designs in Surgery: A Systematic Review

**DOI:** 10.1097/SLA.0000000000005620

**Published:** 2022-07-22

**Authors:** Simone Augustinus, Iris W.J.M. van Goor, Johannes Berkhof, Lois A. Daamen, Bas Groot Koerkamp, Tara M. Mackay, I.Q Molenaar, Hjalmar C. van Santvoort, Helena M. Verkooijen, Peter M. van de Ven, Marc G. Besselink

**Affiliations:** * Department of Surgery, Amsterdam UMC, location University of Amsterdam, The Netherlands; † Cancer Center Amsterdam, Amsterdam, The Netherlands; ‡ Department of Surgery, Regional Academic Cancer Center Utrecht, University Medical Center Utrecht, Cancer Center & St. Antonius Hospital Nieuwegein, Utrecht University, Utrecht, The Netherlands; § Department of Radiation Oncology, Regional Academic Cancer Center Utrecht, University Medical Center Utrecht, Cancer Center, Utrecht University, Utrecht, The Netherlands; ∥ Department of Epidemiology and Data Science, Amsterdam UMC, location Vrije Universiteit van Amsterdam, Amsterdam, The Netherlands; # Department of Surgery, Erasmus Medical Center, Rotterdam, The Netherlands; ¶ Division of Imaging, University Medical Center Utrecht Cancer Center, Utrecht University, Utrecht, The Netherlands; ** Department of Data Science and Biostatistics, Julius Center for Health Sciences and Primary Care, University Medical Center Utrecht, Utrecht, The Netherlands

**Keywords:** randomized controlled trials, registry-based, stepped-wedge, surgical research, trials-within-cohorts

## Abstract

Randomized controlled trials (RCTs) yield the highest level of evidence but are notoriously difficult to perform in surgery. Surgical RCTs may be hampered by slow accrual, the surgical learning curve, and lack of financial support. Alternative RCT designs such as stepped-wedge randomized controlled trials (SW-RCTs), registry-based randomized controlled trials (RB-RCTs), and trials-within-cohorts (TwiCs) may overcome several of these difficulties. This review provides an overview of alternative RCT designs used in surgical research.

**Methods:**

We systematically searched PubMed, EMBASE, and Cochrane Central for surgical SW-RCTs, RB-RCTs, and TwiCs. A surgical RCT was defined as a randomized trial that studied interventions in patients undergoing general surgery, regardless of the affiliation of the corresponding author. Exponential regression analysis was performed to assess time trends.

**Results:**

Overall, 41 surgical RCTs using alternative designs were identified, including 17 published final RCT reports and 24 published protocols of ongoing RCTs. These included 25 SW-RCTs (61%), 13 RB-RCTs (32%), and 3 TwiCs (7%). Most of these RCTs were performed in Europe (63%) and within gastrointestinal/oncological surgery (41%). The total number of RCTs using alternative designs exponentially increased over the last 7 years (*P*<0.01), with 95% (n=39/41) of the total number published within this time frame. The most reported reasons for using alternative RCT designs were avoidance of contamination for SW-RCTs and generalizability of the trial population for RB-RCTs and TwiCs.

**Conclusions:**

Alternative RCT designs are increasingly used in surgical research, mostly in Europe and within gastrointestinal/oncological surgery. When adequately used, these alternative designs may overcome several difficulties associated with surgical RCTs.

Randomized controlled trials (RCTs) provide the highest level of evidence in clinical practice.^1^ However, surgical RCTs are notoriously difficult to perform, mainly due to poor recruitment, patients dropout in the control arm, and high costs.^2^–^4^ These problems are quite common, resulting in 1 in 5 surgical RCTs being discontinued early and 1 in 3 completed surgical RCTs remaining unpublished.^5^ For surgical research specifically, additional problems occur due to surgical learning curves, poor generalizability of the trial population, problems with blinding, and difficulties with randomization in life-threatening situations.^6^ Thereby, surgical RCTs appear to have moderate impact on daily surgical practice with only 47% of surgeons adhering to recommendations of specific RCTs in clinical practice.^7^ In recent years, several alternative RCT designs have been introduced such as stepped-wedge randomized controlled trials (SW-RCTs), registry-based randomized controlled trials (RB-RCTs), and cohort-multiple RCTs, also called trials-within-cohorts (TwiCs), which address several of these problems (Table 1).^8^–^10^

**TABLE 1:** Overview of RCT Designs

	Informed Consent	Randomization	Data Collection
Classic RCT	Before enrollment in the trial (all patients)	Individual randomization and informed consent of all patients	Through (electronic) case report forms specific for the trial
SW-RCT	Depending on the intervention, individual informed consent can be waived due to clusters being randomized	Clusters switch from standard care to intervention at time points. The order in which clusters switch from standard care to intervention is randomized	According to conventional RCT design, or an existing registry or prospective cohort is used in which patient data are routinely registered
RB-RCT	According to conventional RCT design	According to conventional RCT design	An existing registry is used in which patient data are routinely registered
TwiCs	Broad informed consent for data collection and TwiCs before enrollment in cohort. A second informed consent when a patient is randomized to the intervention arm	Patients meeting the inclusion criteria are identified within the cohort and consequently randomized	An existing registry or prospective cohort is used in which patient outcome data are routinely collected

In SW-RCTs, the intervention is sequentially rolled-out and clusters, such as hospitals or hospital wards, switch from standard care to the intervention at different times in a randomized order. Patient inclusion continues throughout the study period so that each cluster contributes to both the control and intervention groups.^11^ In RB-RCTs, an existing prospective patient registry is used. Randomization takes place according to the conventional RCT design and data are collected in the existing registry.^12^ In TwiCs, an existing prospective cohort is used in which outcome measures are collected. Eligible patients are identified from the cohort and consequently randomized for a new intervention or to continue standard care. Only patients randomized to the new intervention are asked for a (second) informed consent. Patients randomized to the comparator arm are not informed and continue standard care.^8^

A systematic review of the experience and use of these alternative RCT designs in surgical research is lacking. Furthermore, the value and suitability of innovative trial designs in surgical research are unclear. The aim of this systematic review is to provide an overview of the experience and use of alternative RCT designs in surgical research, including reported motivations and limitations.

## METHODS

This systematic review was performed in accordance with the Preferred Reporting Items for Systematic Reviews and Meta-Analysis (PRISMA) guidelines.^13^

### Search Strategy and Selection Process

The literature search strategy was developed with an experienced clinical librarian to identify eligible trials in PubMed, EMBASE, and Cochrane Central from inception. The search included various synonyms for the words “stepped-wedge,” “registry-based,” “trial-within-cohort,” and “surgical procedures” (Supplement 1, http://links.lww.com/SLA/E66). Inclusion criteria comprised: (1) final RCT reports and published trial protocols of ongoing RCTs; (2) either SW-RCTs, RB-RCTs, or TwiCs; (3) surgical intervention trials. Exclusion criteria comprised: (1) unpublished trial protocols; (2) conference abstracts; (3) secondary publications of previously published trials; (4) commentaries/letters to the editor; (5) publications in languages other than English.

Two reviewers (S.A. and I.W.J.M.v.G.) independently screened all abstracts for relevance. Subsequently, full-text versions of all relevant studies were reviewed and a final selection was made. Disagreements were resolved by reaching a consensus by a third reviewer (M.G.B. and P.M.v.d.V.). The methodological quality was evaluated by 2 independent reviewers (S.A. and I.W.J.M.v.G.) using the Cochrane Risk of Bias 2 tool (RoB-2) for RCTs. Methodological quality was only assessed for final RCT reports, not for protocols of ongoing RCTs, as not all domains could be evaluated for protocols.^14^ Adherence to reporting guidelines specific for the design was reported for SW-RCTs and defined as specific reference to the CONSORT extension for either cluster randomized trials or stepped-wedge designs.^15^,^16^ This was only assessed for SW-RCTs published after 2010, the year in which this extension was published. Adherence to reporting guidelines was not assessed for RB-RCTs and TWICs as a CONSORT extension for these trials was only published in 2021.^17^

### Definitions and Extraction of Data

The definitions used and study data extracted can be found in Supplement 2 (http://links.lww.com/SLA/E67).

### Statistical Analysis

Descriptive analyses were used to summarize study characteristics per design. Characteristics were summarized as frequencies with proportions for binary or categorical variables, or as mean with SD or median with interquartile range or range for continuous variables as appropriate. The number of innovative RCTs per type of design per year was calculated and depicted graphically. Exponential regression analysis was performed to assess the change in the number of published studies over the years 2015 to 2021. Reported motivations for use and limitations were ordered according to the number of times reported, separate for each type of design.

## RESULTS

**FIGURE 1:**
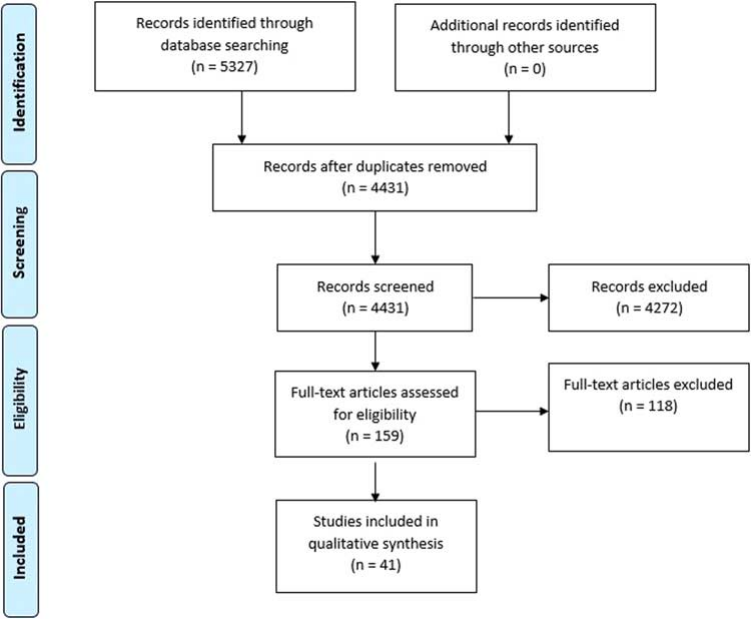
PRISMA flow diagram.

The literature search was performed on February 3, 2022, and identified 4431 articles. After title and abstract screening, full-text screening of 159 articles was performed. In total, 41 surgical RCTs met the inclusion criteria (Fig. 1).

### Overview

**FIGURE 2:**
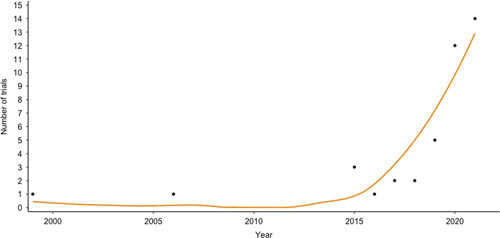
Time trend in published surgical RCTs and RCT protocols with alternative designs.

Among the 41 included surgical RCTs, 17 were final RCT reports, and 24 were published protocols of ongoing RCTs (Supplement 3, http://links.lww.com/SLA/E68). Overall, 25 trials were SW-RCTs (61.0%), 13 were RB-RCTs (31.7%), and 3 were TwiCs (7.3%). Seven out of 25 SW-RCTs (28.0%) and all 3 TwiCs (100%) used data from existing registries for their data collection. Most trials were initiated in Europe (n=26, 63.4%), followed by the United States (n=10, 24.4%), Canada (n=3, 7.3%), and New Zeeland (n=2. 4.9%). For individual countries, most trials were initiated in The Netherlands (n=12, 29.3% in all countries and 48% in Europe). Trials were published between 1999 and 2021, of which the vast majority (95.1%) were published since 2015. Since 2015, the volume of innovative trial designs increased exponentially with time (Fig. 2, *R* ^2^=0.80, *F*_1,6_=24.7, *P*<0.01). Most trials were performed in the field of gastrointestinal/oncological surgery (41.5%). For SW-RCTs, the majority of trials investigated nontherapeutic interventions (88.0%), whereas for RB-RCTs (76.9%) and TwiCs (100%) the majority investigated therapeutic interventions. Median duration of recruitment was 28 months (interquartile range: 19.2–45 months) for published studies. Of the unpublished trials, current recruitment status was known for 22 of 24 trials. Most of these studies had finished recruiting patients (n=14, 63.6%), 6 studies (26.1%) were currently recruiting patients, and only 2 studies (9.1%) were stopped prematurely, of which 1 (4.2%) was stopped because of slow accrual (Supplement 4, http://links.lww.com/SLA/E69).

### Risk of Bias

Risk of bias was considered low for 10 out of 17 final RCT reports (58.8%), although some concerns were reported^18^–^29^ (Table 2). Concerns were mostly related to assessors not being blinded for the intervention, although it was acknowledged that this was unlikely to influence the results. Of the 24 final RCT reports and protocols using the SW-RCT design (published after 2010), only 6 (25.0%) reported the use of a CONSORT extension.

**TABLE 2:**
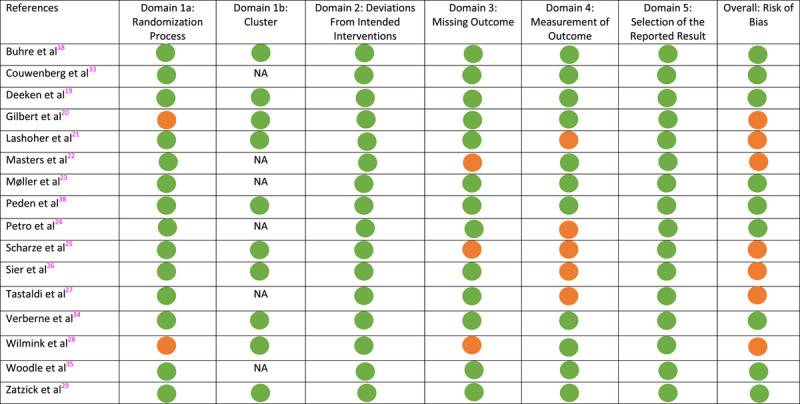
Risk of Bias of 17 Published Final Reports of RCTs With Alternative Designs

Black: low risk of bias; gray: some concerns.

NA indicates not applicable.

### Reported Motivations

The most reported reason for choosing the SW-RCT design was the minimization of contamination (for all reported motivations, Supplement 5, http://links.lww.com/SLA/E70). For example, one trial implemented a national histopathology service to aid the selection of better quality kidneys. If this trial would randomize individual kidneys with or without the option to use the histopathology service, the extra information for only some kidneys would probably “contaminate” (ie, change) acceptance practice of kidneys offered without it.^30^

The most reported reason for using a RB-RCT design, also when this was incorporated in a SW-RCT or TwiCs study, was improved generalizability of the trial (for all reported motivations, Supplement 6, http://links.lww.com/SLA/E71). For example, by facilitating the inclusion of a relatively large group of patients and embedding the research question in the clinical practice (ie, the registry).^31^,^32^

The most reported reason for using TwiCs, besides the advantages of a registry, was avoidance of disappointment bias. As only patients randomized to the intervention group are made aware of the randomization result, patients will not be disappointed and dropout of the study due to randomization into the control arm.^33^

### Reported Limitations

For SW-RCTs, the most common limitation was confounding of treatment effect by time. (for all reported limitations, Supplement 7, http://links.lww.com/SLA/E72). For example, 1 SW-RCT implemented an intensified follow-up schedule to detect recurrence after curative colorectal cancer treatment. However, as incidence of recurrence tends to change over time during follow-up, it can never be known whether the observed effects are completely due to the intervention.^34^

For RB-RCTs, the most frequently reported limitation was that the trial was limited to the variables recorded in the registry and the patient population included in the registry (for all reported limitations, Supplement 8, http://links.lww.com/SLA/E73). For example, in 1 RB-RCT trial available long-term outcomes were limited to allograft failure and patient death, but outcomes of kidney function, metabolic complications, cardiovascular event, and infections were also of interest but not registered.^35^

For TwiCs, selective patient refusal was mentioned as a limitation. Patients allocated to the intervention arm have to sign a second informed consent but may refuse the intervention, which dilutes the effect estimates in intention-to-treat analyses.^33^

## DISCUSSION

This first systematic review of alternative RCT designs in surgical research identified 41 RCTs, including 17 published and 24 ongoing trials. Of these, the majority were SW-RCTs (61.0%), 13 were RB-RCTs (31.7%), and 3 were TwiCs (7.3%). Most trials were initiated in Europe and performed within gastrointestinal/oncological surgery. The vast majority of alternative RCTs were published after 2015 demonstrating that their use is increasing rather rapidly.

The alternative RCT designs were introduced to overcome challenges encountered within classic surgical RCTs. One of the biggest challenges is poor patient accrual, leading to long inclusion times and high costs.^5^ The most effective and most widely used design for overcoming this challenge seems to be SW-RCT. In SW-RCTs, whole clusters are being randomized, often without the requirement of individual informed consent.^11^ Also, RB-RCTs may improve patient accrual as they allow for broader inclusion criteria, and recruiting from multiple providers and regions. Thereby, centers are generally more willing to participate as costs are minimal when data collection is already ongoing within the registry.^36^ In TwiCs, the dropout of patients randomized to the control group is almost nonexistent.^37^ Therefore, each of these alternative trial designs potentially increase the accrual rate. This is supported by the relatively short recruitment period of 28.0 months for the published studies included and two third of the ongoing RCTs already having completed recruitment (63.6%). However, these results should be interpreted with caution because of potential selection bias. Authors may be more likely publish protocols of studies expected to finish recruitment. Unpublished protocols of ongoing RCTs are not included in this review.

When deciding between designing a “classical” or an “alternative” RCT design, surgeon-scientists should consider which specific challenges are relevant for their study on a case-by-case basis. Problems of the learning curve and blinding are not solved by the alternative designs. Guidelines should be developed assessing the suitability of the alternative trial designs for each setting, for example, by performing a Delphi study including surgeons and epidemiologists with expertise on the topic. Below we further explain the merits per alternative design with examples (summarized in Table 3).

**TABLE 3:** Summary of the 3 Alternative Designs

Trial Design	Settings Where Suitable	Textbook Example	Main Motivation(s)	Main Limitation(s)	Points of Consideration
SW-RCT	Strong believe that the new intervention is beneficial Implement of the intervention across all clusters is desirable	Evaluation of a national quality improvement program that implements a care pathway for emergency abdominal surgery^26^	Minimization of contamination High participation rates as all clusters are automatically exposed to the new intervention	Confounding of treatment effect by time Statistically complex (sample size calculation and adjustment for clusters)	Involve an experienced statistician to improve (methodological) quality
RB-RCT	Pragmatic trails requiring large numbers of patients representative of a real-world clinical population	Screening trial for AAA randomized 12,639 patients to either an abdominal ultrasound scan or the control group with outcomes collected from Danish registries^29^	Increased generalizability of the trial population, as inclusion criteria for registries are generally less restrictive than for standard trial	Lack of detailed data Quality of registry data	Add trial-specific variables temporarily to the registry Optimize data quality
TwiCs	Dropout in standard RCTs is expected to high for subjects randomized to the control arm Multiple new treatments for the same conditions are to be evaluated (almost) simultaneously	Trial investigates how many stage II colorectal cancer patients with ctDNA will accept adjuvant chemotherapy and whether this reduces the risk of recurrence. Data are collected and patients are identified in the Prospective Dutch Colorectal Cancer Cohort (PLCRC)^32^	Increase of inclusion rates by avoidance of dropout due to disappointment bias More efficient when large number of potential controls are available	Patients refusing participation in the intervention arm may be high. This can dilute the outcome	Take selective refusal into account in sample size calculation to avoid loss of statistical power Requires good explanation of the design to researchers and patients

Table is based on the motivations and limitations reported in this review combined with the available literature.

AAA indicates aortic abdominal aneurysm; ctDNA, circulating tumor DNA.

SW-RCTs are best suited for trials in which there is a strong evidence or belief that the new intervention is beneficial and a decision has already been made to implement this intervention across all clusters.^41^ This scenario mostly applies to nontherapeutic interventions (88.0% of SW-RCTs in this review investigated nontherapeutic interventions) such as quality improvement programs or best practice implementations. Notably, these programs or pathways in themselves may include specific interventions. A textbook example is the evaluation of a national quality improvement program that implements a care pathway for emergency abdominal surgery.^38^ The main advantage reported in the present systematic review was minimization of contamination and SW-RCTS are associated with high participation rates as all clusters are automatically exposed to the new intervention. Whereas the main limitation reported was confounding of treatment effect by time. A previous review focusing specifically on statistical methods in SW-RCTs indicates that only 33% of SW-RCTs corrected for time effects. Since the proportion of subjects in SW-RCT that receive the new intervention increases over time and outcomes generally depend on time or confounding factors changing over time, a correction for time is essential in SW-RCTs^42^,^43^ Within these reviews, additional statistical problems for SW-RCTs are mentioned (which are not reported in the present review), only 75% of SW-RCTs reported a sample size calculation and only 73% adjusted for clustering.^42^,^43^ Therefore, when performing SW-RCTS we advise to involve an experienced statistician to improve (methodological) quality.

RB-RCTs are best served for pragmatic trials requiring large numbers of patients representative of a real-world clinical population to show effectiveness on outcomes collected in routine care.^9^ As a textbook example, the randomized single-center mass screening trial for abdominal aortic aneurysm, randomized 12,639 patients to either an abdominal ultrasound scan or the control group with outcomes collected from Danish registries.^39^ The advantages and limitations of surgical RB-RCTs in this review are in agreement with articles evaluating the RB-RCTs in general.^9^,^36^,^44^ RB-RCTs primarily overcome the problem of poor generalizability of the trial population, as registries are generally less restrictive than standard trial inclusion criteria. Important limitations mainly concern the lack of detailed data and internal validity. Only in 11.3% of the RB-RCTs, the quality of the registry data is mentioned.^36^ Therefore, when performing a surgical RB-RCT, the potential lack of quality of the registry data should be carefully weighed against the advantage of efficient data collection, or measures should be taken to improve the quality. Some registries may be able to add trial-specific variables temporarily to the registry.

The TwiCs design is best used when dropout in standard RCTs is expected to high for subjects randomized to the control arm and when multiple new treatments for the same condition are expected to be evaluated (almost) simultaneously.^45^ As a textbook example, the MEDOCC-CrEATE study investigates how many stage II colorectal cancer patients with detectable circulating tumor DNA after surgery will accept adjuvant chemotherapy and whether this reduces the risk of recurrence in these patients.^40^ Data are collected and patients are identified in the Prospective Dutch Colorectal Cancer Cohort (PLCRC). Main advantages reported in this review are conform literature, namely the increase of inclusion rates by reducing dropout due to disappointment bias and more efficient data collection due to the large number of potential controls.^45^,^46^ In terms of limitations, the number of patients refusing participation in the intervention arm is usually higher than in a conventional RCT due to the fact that additional informed consent is obtained after randomization.^46^,^47^ However, given that recruitment is more easy, this most likely weights up to the patients refusing participation in the intervention arm.^48^ Thereby, the percentage of patients accepting the intervention can reflect the acceptance of patients in current clinical practice^46^. However, selective refusal should be taken into account in the sample size calculation to avoid low statistical power.^48^ Furthermore, the study design should be explained well to both researchers and patients.

The use of reporting guidelines, including the Consolidated Standards of Reporting Trials (CONSORT) statement, improves reporting quality and enables adequate assessment of RCTs.^49^ In 2010, a CONSORT extension for SW-RCTs has been published, and in 2021 an extension for trials was conducted using cohorts and routinely collected data (CONSORT-ROUTINE).^15^,^17^ In this review, only 25% of SW-RCTs mention the use of the CONSORT extension, and CONSORT-ROUTINE was not yet available for the RB-RCTs and TwiCs. Improved use of these CONSORT extensions can be considered an essential step to increase the quality of studies using alternative trial designs. A next step would be to incorporate these CONSORT extensions into a decision-making and risk assessment tool, such as has been done in the RoB-2 tool for cluster-RCTs.^50^ In this risk of bias that has been evaluated, 7 out of 10 final RCT reports indicate some concerns, of which 5 were related to outcome measure. This was due to assessors not being blinded for the intervention, although it was acknowledged that this was unlikely to influence the results. This is comparable with regular RCT designs, and not specific for alternative RCT designs as in a review evaluating all surgical RCTs adequate generation and concealment of allocation seem to be a problem in 47% to 50% of RCTs.^51^

This study has several limitations. First, most included studies were published trial protocols of ongoing RCTs. In these protocols, only the a priori motivations and limitations were reported, and additional motivations and limitations (including performance) may become apparent at a later stage. A related limitation is that risk of bias could not be evaluated for the protocols. Nevertheless, the published protocols of ongoing RCTs do give an overview of the use of the trials designs, and often in the published protocols, methodology is described more elaborate giving us more information about the motivations and limitations of the designs. Second, the RoB-2 tool for includes an extension for cluster-RCTs, but extensions for SW-RCTs, RB-RCTs, and TwiCs are not yet incorporated. As these extensions were not taken into account in the risk of bias evaluation, specific details regarding these designs could not be assed. This might give an underestimation of bias of the studies as, for example, the quality of the registries and cohorts are not evaluated. Third, only 3 alternative trial designs are included, but more alternative trial designs are being used, such as trials using a patient preference design.^52^ Fourth, publication bias may have occurred because of possible limitations arisen during the trial being underreported. Fifth, the number of alternative RCTs may still be considered limited with 17 published RCTs across 3 designs. For instance, only 1 published TwiCs could be included. With more alternative RCTs becoming available the overall assessment of the specific merits could therefore still change (Supplement 9, http://links.lww.com/SLA/E74).

To conclude, the use of alternative trial designs within surgical research is increasing, especially over the last years. If adequately used, these innovative trial designs provide the opportunity to overcome specific difficulties associated with surgical research. However, as these designs also have their limitations, the surgeon should decide on a case-by-case basis which design is best suitable for each specific setting and use the CONSORT extensions.

## Supplementary Material

SUPPLEMENTARY MATERIAL

## DISCUSSANT

**Inne H.M. Borel Rinkes (Utrecht, The Netherlands)**

I would like to thank the ESA for the privilege of discussing this very interesting paper. Dr. Augustinus and her colleagues are to be complimented for this overview of new randomized control trial designs. These are much desired because of the problems encountered when performing RCTs, particularly in the field of surgery. The authors described the three alternatives elegantly.

My first question regards the adherence to the trial results. We published a paper on this topic, which was presented in Madrid two years ago, and less than half of the responding surgeons admitted that they would comply with these results; more than half would not. Do you think that introducing these new trials will encourage surgeons to adopt their results, or change their behaviors?

Second, you’ve based your review on trial descriptions in more than half of your RCTs (23 out of 41; only 18 involved true RCT results). Perhaps, this is because they are new trial designs, but is that sufficient to base your conclusions on?

Similarly, you showed one motivation and one limitation per trial design, based on what the authors spontaneously reported. However, despite them being good motivations and limitations, there must be more. Perhaps, it would have been better to interview these authors directly, in order to really understand whether there were other reasons for their motivations.

Third, although the trial designs do appear to speed up or facilitate trial completion, they also each have their own specific problems. You have alluded to some concerns regarding a lack of quality criteria. So, the question remains: will the novel designs ultimately provide quality levels like classic RCTs? What should we do to achieve this?

Finally, the step-wedge trials have all focused on non-surgical interventions. Would surgical interventions be suitable for these trial designs as well?

**Response from Simone Augustinus (Amsterdam, The Netherlands)**

Thank you very much for the compliments and questions. To answer your first question, which is perhaps the most important one. I think that the step-wedge trial design can best solve this. In this design, which could also involve low-complex surgical interventions, the intervention is often implemented in all clusters (hospitals and patients) during the trial. Hereafter, if the intervention is proven to be effective, everyone will usually continue working with these interventions. In particular, registry-based trials improve the generalizability of trial results. In the survey you mention, the most commonly reported reason for not adhering was the use of a highly selective population. By incorporating trials into registries, the results can be more generalized and that might overcome the issue that people have when implementing them within a clinical practice. This is the same for Trials Within Cohorts (TWiCs), where patients are asked to sign a consent form once they are informed of the intervention that they may become eligible for. So, you can really see whether patients want the intervention in clinical practice, as it is only offered to patients who subsequently randomize for the intervention arm. These are some of the ways that the different trial designs can improve adherence.

Regarding the protocols, clearly not everything is incorporated, but generally, the protocols incorporate extensive information on the motivations and limitations. However, I think that now is the right time to present these results, as we see that these trial designs are increasingly used. Therefore, we do need to pay attention to what the observed limitations are and whether we should perform more research to adequately interpret these results.

I think this also answers your other question regarding the next steps to make sure we have all the motivations and limitations. We could have an expert survey, including surgeons and statisticians with expertise on this topic, to bring additional perspectives.
