# Supplementary material for: Alternative Randomized Trial Designs in Surgery: A Systematic Review
Source: Ann Surg. 2022 Jul 22;276(5):753–60. doi: 10.1097/SLA.0000000000005620 (PMC9534057; doi:10.1097/SLA.0000000000005620)
Supplement: SUPPLEMENTARY MATERIAL [file sla-276-0753-s001.docx]

**Supplement 1.** Search strategy

| **Database** | **Search** | **Results** |
| --- | --- | --- |
| **Pubmed** | (registry based randomized controlled trial*[tiab] OR registry based randomised controlled trial*[tiab] OR registry based randomized trial*[tiab] OR registry based randomised trial*[tiab] OR registry based randomised clinical trial*[tiab] or registry based randomized clinical trial*[tiab] OR R-RCT*[tiab] OR registry embedded clinical trial*[tiab] OR registry trial*[tiab] OR pragmatic trial*[tiab] OR pragmatic clinical trial*[tiab] OR SW-CRT*[tiab] OR stepped-wedge[tiab] OR cmRCT*[tiab] OR cohort multiple randomized trial*[tiab] OR cohort multiple randomised trial*[tiab] OR TwiCs[tiab] OR TwiC[tiab] OR trials within cohort*[tiab] OR trial within cohort*[tiab] OR staged introduction[tiab] OR phased implementation[tiab] OR staggered implementation[tiab] OR phased recruitment[tiab] OR stepwise recruitment[tiab] OR one way crossover[tiab]) AND ("General Surgery"[Mesh] OR "Surgical Procedures, Operative"[Mesh] OR surger*[tiab] OR surgic*[tiab] OR chirur*[tiab] OR cirugia[tiab] OR kirurg*[tiab] OR surger*[ad] OR surgic*[ad] OR chirur*[ad] OR cirugia [ad] OR kirurg* [ad]) | 1087 |
| **EMBASE** | #1 (registry based randomized controlled trial* or registry   based randomised controlled trial* or registry-based   randomised clinical trial* or registry-based   randomized clinical trial* or registry embedded clinical   trial* or registry trial* or registry-based randomized   trial* or registry-based randomised trial* or RB-RCT*   or pragmatic trial* or pragmatic clinical trial* or R-  RCT* or SW-CRT* or stepped-wedge or cmRCT* or   cohort multiple randomized trial* or cohort multiple   randomised trial* or TwiCs or TwiC or trials within   cohort* or trial within cohort*).ti,ab,kw  #2 "randomized controlled trial (topic)"/ and register/  #3 (registry based adj3 (randomized controlled trial* or   randomised controlled trial* or randomised clinical   trial* or randomized clinical trial*)).ti,ab,kw.  #4 ("staged introduction" or "phased implementation" or   "staggered implementation" or "phased recruitment"   or "stepwise recruitment" or "one way   crossover").ti,ab,kw.  #5 1 or 2 or 3 or 4  #6 exp surgery/ or surgery.fs.  #7 (surger* or surgic* or chirur* or cirugia or   kirurg*).ti,ab,kw.  #8 6 or 7  #9 5 and 8 | 1277 |
| **Cochrane Libarary** | #1 (registry based randomized controlled trial* or   registry based randomised controlled trial* or registry-  based randomised clinical trial* or registry-based   randomized clinical trial* or registry embedded clinical   trial* or registry-based randomized trial* or registry-  based randomised trial* or RB-RCT* or R-RCT* or SW-  CRT* or stepped-wedge or cmRCT* or cohort multiple   randomized trial* or cohort multiple randomised trial*   or TwiCs or TwiC or trials within cohort* or trial within   cohort*):ti,ab,kw  #2 (registry based near/2 (randomized controlled trial*   or randomised controlled trial* or randomised clinical   trial* or randomized clinical trial*)):ti,ab,kw  #3 ("staged introduction" or "phased implementation" or   "staggered implementation" or "phased recruitment"   or "stepwise recruitment" or "one way   crossover"):ti,ab,kw  #4 #1 or #2 or #3  #5 (surger* or surgic* or chirur* or cirugia or   kirurg*):ti,ab,kw  #6 #4 and #5 in Trials | 2963 |
| **Total** |  | 5327 |
