# Supplementary material for: Alternative Randomized Trial Designs in Surgery: A Systematic Review
Source: Ann Surg. 2022 Jul 22;276(5):753–60. doi: 10.1097/SLA.0000000000005620 (PMC9534057; doi:10.1097/SLA.0000000000005620)
Supplement: SUPPLEMENTARY MATERIAL [file sla-276-0753-s002.docx]

**Supplement 2**. Definitions and extraction of data

*Definitions*
A surgical RCT was defined as a randomized trial that studied interventions in patients undergoing general surgery, regardless of the affiliation of the corresponding author. General surgery was defined as gastrointestinal/oncological, trauma, vascular, thoracic, breast, pediatric, and abdominal transplant surgery. An intervention in patients undergoing surgery was defined as any perioperative intervention including surgical techniques, (neo-)adjuvant therapy, and other interventions in the clinical surgical ward or outpatient clinic. If an intervention was performed in multiple clinical wards, at least one author from the surgical department had to be listed as a collaborator, and at least 50% of patients to be included at the surgical department. An intervention was considered therapeutic when it assessed the effect of specific treatment (e.g., surgical procedure or medication). Examples of non-therapeutic interventions are quality improvement programs and quality control algorithms.

*Extraction of trial data*

Study characteristics were extracted (i.e. year of publication, type of publication, country, impact factor, type of surgical specialty, aim of the trial, and type of intervention) together with reported motivations for and limitations of the chosen trial design. Authors of yet unpublished trials were contacted to enquire about the current status of the trial.
