# Supplementary material for: Alternative Randomized Trial Designs in Surgery: A Systematic Review
Source: Ann Surg. 2022 Jul 22;276(5):753–60. doi: 10.1097/SLA.0000000000005620 (PMC9534057; doi:10.1097/SLA.0000000000005620)
Supplement: SUPPLEMENTARY MATERIAL [file sla-276-0753-s003.docx]

**Supplement 3A.** Overview of included SW-RCTs

| **Auteur** | **Type of publication** | **Country** | **Impact factor**** | **Recruitment period (months)***** | **Type of surgical specialty** | **Aim** | **Type of intervention (therapeutic / non-therapeutic)** |
| --- | --- | --- | --- | --- | --- | --- | --- |
| Anderson, 2020* | Protocol | USA | 2.1 | NA | Different types of surgery combined | Determine if using optimized statistical process control charts for SSI surveillance and feedback lead to a reduction in SSI rates compared to traditional surveillance | Non-therapeutic |
| Ayorinde, 2019* | Protocol | United Kingdom | 2.7 | NA | Transplant surgery | Implementation of a national, 24 hours, digital histopathology service to improve outcomes of kidney transplantation | Non-therapeutic |
| Buhre, 2021^40^ | Final RCT report | the Netherlands | 12.9 | 22.0 | Different types of surgery combined | Impact of standardized postoperative anesthesia visits after elective non-cardiac surgery on 30-day mortality | Non-therapeutic |
| Deeken, 2021^41^ | Final RCT report | Germany | 14.8 | 16.7 | Different types of surgery combined | Effect of multifaceted prevention intervention on postoperative delirium after major surgical procedures | Non-therapeutic |
| De Mik, 2020* | Protocol | the Netherlands | 3.2 | NA | Vascular surgery | Effectiveness and implementation of decision support tools to improve shared decision-making during vascular surgical consultations | Non-therapeutic |
| Douillet, 2021* | Protocol | France | 2.7 | NA | Trauma surgery | Demonstrate the safety of tromboprophylaxis based on the TRiP(cast) score in patients with lower limb trauma requiring immobilization in the emergency department | Therapeutic |
| Gilbert, 2021^42^ | Final RCT report | France | 4.6 | 38.0 | Gastrointestinal/  Oncological surgery | Impact of geriatric intervention on the screening and management of undernutrition in elderly patients scheduled for colon cancer surgery | Non-therapeutic |
| Grossi, 2018* | Protocol | United Kingdom | 2.1 | NA | Gastrointestinal/  Oncological surgery | Clinical efficacy of laparoscopic ventral mesh rectopexy in adults with chronic constipation | Therapeutic |
| Lashoher, 2017^43^ | Final RCT report | France | 3.4 | 19.2 | Trauma surgery | Implementation of the WHO trauma care checklist to improve care for injured patients in low-, middle-, and high-income countries | Non-therapeutic |
| Mackay, 2020* | Protocol | the Netherlands | 2.1 | NA | Gastrointestinal/  Oncological surgery | Impact of nationwide enhanced implementation of best practices to improve survival in pancreatic cancer care | Non-therapeutic |
| Malone, 2021* | Protocol | USA | 7.3 | NA | Pediatric surgery | Assess the impact of two potential strategies to de-implement unnecessary post-operative antibiotic use in children on post-operative antibiotic prescribing in low risk surgical cases | Non-therapeutic |
| Noordman, 2018* | Protocol | the Netherlands | 4.4 | NA | Gastrointestinal/  Oncological surgery | Effectiveness of active surveillance versus neoadjuvant chemoradiotherapy plus surgery for esophageal cancer | Therapeutic |
| Peden, 2019^26^ | Final RCT report | USA | 79.3 | 19.5 | Gastrointestinal/  Oncological surgery | Effectiveness of national quality improvement program to implement a care pathway for emergency abdominal surgery | Non-therapeutic |
| Pagano, 2021* | Protocol | Italy | 2.7 | NA | Gastrointestinal/  Oncological surgery | Compare standard perioperative management and ERAS on length of hospital stay in patients undergoing colorectal cancer surgery | Non-therapeutic |
| Pourrat, 2021* | Protocol | France | 2.1 | NA | Transplant surgery | Implementation of a personalized pharmaceutical plan to increase adherence to immunosuppressive drugs and increase graft survival. | Non-therapeutic |
| Raval, 2020* | Protocol | USA | 2.1 | NA | Pediatric surgery | Effectiveness of implementation of a perioperative enhanced recovery protocol for children undergoing surgery | Non-therapeutic |
| Schwarze, 2020^47^ | Final RCT report | USA | 14.8 | 29.0 | Different types of surgery | To compare effectiveness of a question prompt list versus usual care on patient engagement and well-being among older patients. | Non-therapeutic |
| Sier, 2017^48^ | Final RCT report | the Netherlands | 2.8 | 26.0 | Gastrointestinal/  Oncological surgery | Home visits as part of a new care pathway in ostomy patients to assess stoma-related complications and improve quality of life | Non-therapeutic |
| Smits, 2020* | Protocol | the Netherlands | 2.1 | NA | Gastrointestinal/  Oncological surgery | Implementation of an algorithm for early detection and minimally invasive management of pancreatic fistula in patients after pancreatic resection | Non-therapeutic |
| Straatman, 2015* | Protocol | the Netherlands | 2.1 | NA | Gastrointestinal/  Oncological surgery | Effect of standardized postoperative quality control algorithm on postoperative morbidity and mortality after major abdominal surgery | Non-therapeutic |
| Van der Sluijs, 2020* | Protocol | the Netherlands | 4.4 | NA | Trauma surgery | Assess impact of Trauma Triage App to aid Emergency Medical Services professionals in field triage | Non-therapeutic |
| Verberne, 2015^22^ | Final RCT report | the Netherlands | 3.9 | 45.0 | Gastrointestinal/  Oncological surgery | Value of follow-up including CEA measurement and CEA-triggered imaging for detecting recurrent disease in colorectal cancer patients | Non-therapeutic |
| Weller, 2020* | Protocol | New Zeeland | 2.7 | NA | Different types of surgery | Effect of multidisciplinary simulation-based team training for surgical teams to improve teamwork and surgical patient outcomes. | Non-therapeutic |
| Wilmink, 1999^50^ | Final RCT report | United Kingdom | 4.3 | 72.0 | Vascular surgery | Estimate the influence of screening on AAA on incidence and mortality of ruptured AAA | Non-therapeutic |
| Zatzick, 2021^52^ | Final RCT report | USA | 14.8 | 46.0 | Trauma surgery | Assess effectiveness and implementation of a stepped collaborative care intervention targeting PTSD and comorbidity for injured patients | Non-therapeutic |

NA: not applicable, SW-RCTs: stepped-wedge randomized controlled trials, USA: united states of America, SSI: surgical site infection, WHO: world health organization, ERAS: enhanced recovery after surgery, AAA = abdominal aortic aneurysm, CEA: cardioembrynoic antigen. *Reference of published protocols are depicted in Supplement 9. **As reported in 2020.***Based on dates reported in final RCT reports (dd-mm-yyy), when day is not reported it is noted as “01”, when months is not reported it is noted as “01”.

**Supplement 3B.** Overview of included RB-RCTs

| **Auteur** | **Type of publication** | **Country** | **Impact factor*** | **Recruitment period (months)***** | **Type of surgical specialty** | **Aim** | **Type of intervention (therapeutic / non-therapeutic)** |
| --- | --- | --- | --- | --- | --- | --- | --- |
| Apte, 2020* | Protocol | Canada | 2.7 | NA | Gastrointestinal/  Oncological surgery | No preparation versus preoperative oral antibiotics to reduce surgical site infection rate in elective colon surgery | Therapeutic |
| Brajcich, 2021* | Protocol | USA | 1.2 | NA | Gastrointestinal/  Oncological surgery | Evaluating piperacillin-tazobactam compared with cefoxitin for SSI prevention following pancreatoduodenectomy | Therapeutic |
| Collins, 2020* | Protocol | New Zeeland | 2.1 | NA | Transplant surgery | Effect of Plasma-Lyte 148 versus 0.9% saline on delayed graft function in kidney transplantation | Therapeutic |
| Eslami, 2015* | Protocol | USA | 1.4 | NA | Vascular surgery | Effectiveness of open versus endovascular repair of asymptomatic popliteal artery aneurysms | Therapeutic |
| Hedberg, 2019* | Protocol | Sweden | 2.2 | NA | Gastrointestinal/  Oncological surgery | Roux-en-Y gastric bypass versus sleeve gastrectomy for severe obesity. | Therapeutic |
| Lindholt, 2006^29^ | Final RCT report | Denmark | 7.1 | 48.0 | Vascular surgery | Effect of mass screening trial for abdominal aortic aneurysm | Non-therapeutic |
| Masters, 2021^44^ | Final RCT report | United Kingdom | 4.3 | 7.0 | Trauma surgery | Standard wound management versus negative-pressure wound therapy on deep infections in the treatment of adult patients following hip fracture surgery | Therapeutic |
| Møller, 2019^45^ | Final RCT report | Denmark | 22.1 | 17.0 | Vascular surgery | Effect of a protocol aiming to restrict RBC transfusion in vascular surgery | Non-therapeutic |
| Petro, 2021^46^ | Final RCT report | USA | 14.8 | 28.0 | Hernia surgery | Determine whether robotic approach to ventral hernia repair with intraperitoneal mesh would result in less postoperative pain compared to laparoscopic | Therapeutic |
| Renz, 2021* | Protocol | Germany | 2.7 | NA | Gastrointestinal/  Oncological surgery | Asses pylorus preservation or pylorus resection in pancreatoduodenectomy on postoperative delayed gastric emptying | Therapeutic |
| Tastaldi, 2019^49^ | Final RCT report | USA | 4.7 | 11.0 | Hernia surgery | Telescopic dissection versus balloon dissection for laparoscopic totally extraperitoneal inguinal hernia repair | Therapeutic |
| Woodle, 2021^51^ | Final RCT report | Canada | 14.8 | 37.0 | Transplant surgery | Compare long-term kidney transplant outcomes of patients randomized to continue or withdraw corticosteroids | Therapeutic |
| Yohanna, 2021* | Protocol | Canada | 1.0 | NA | Transplant surgery | Determine if a quality improvement intervention in chronic kidney disease enables more patients with to complete more steps towards receiving a kidney transplant | Non-therapeutic |

RB-RCT: registry based randomized controlled trial, USA: united states of America. SSI: surgical site infection *Reference of published protocols are depicted in Supplement 9. **As reported in 2020. ***Based on dates reported in final RCT reports (dd-mm-yyy), when day is not reported it is noted as “01”, when months is not reported it is noted as “01”.

**Supplement 3C.** Overview of included TwiCs

| **Auteur** | **Type of publication** | **Country** | **Impact factor**** | **Recruitment period (months)***** | **Type of surgical specialty** | **Aim** | **Type of intervention (therapeutic / non-therapeutic)** |
| --- | --- | --- | --- | --- | --- | --- | --- |
| Couwenberg, 2020^21^ | Final RCT report | the Netherlands | 7.0 | 46.0 | Gastrointestinal/ Oncological surgery | Effect of external radiation boost to the tumor before chemoradiation on pathologic clinical tumor response in locally advanced rectal carcinoma | Therapeutic |
| Couwenberg, 2016* | Protocol | the Netherlands | 2.1 | NA | Gastrointestinal/ Oncological surgery | Impact of retractor sponge-assisted laparoscopic surgery on length of stay and postoperative complications in elective colon surgery | Therapeutic |
| Schraa, 2020* | Protocol | the Netherlands | 4.4 | NA | Gastrointestinal/ Oncological surgery | Acceptance rate of adjuvant chemotherapy and reduction of risk of recurrence in patients with stage II colon cancer and circulating tumor DNA. | Therapeutic |

TwiCs = Trials within Cohorts. *Reference of published protocols are depicted in Supplement 9. **As reported in 2020. ****Based on dates reported in final RCT reports (dd-mm-yyy), when day is not reported it is noted as “01”, when months is not reported it is noted as “01”.*
