# Supplementary material for: Alternative Randomized Trial Designs in Surgery: A Systematic Review
Source: Ann Surg. 2022 Jul 22;276(5):753–60. doi: 10.1097/SLA.0000000000005620 (PMC9534057; doi:10.1097/SLA.0000000000005620)
Supplement: SUPPLEMENTARY MATERIAL [file sla-276-0753-s004.docx]

**Supplement 4.** Status of published protocols

| **Protocols** | **Year start trial*** | **Status**** |
| --- | --- | --- |
| Eslami, 2015 | ? | 6. Other: stopped due to logistical issues |
| Straatman, 2015 | 2015 | 6. Other: stopped due to regional regulations and lack of adequate funds, partly continued as a mono-center study. |
| Couwenberg, 2016 | 2015 | 1. Finished recruiting, manuscript submitted |
| Grossi, 2018 | 2015 | 1. Finished recruiting, manuscript submitted |
| Hedberg, 2019 | 2015 | 3. Currently recruiting, including patients |
| Noordman, 2018 | 2017 | 2. Finished recruiting, currently analysing |
| Ayorinde, 2019 | 2017 | 2. Finished recruiting, currently analysing |
| Anderson, 2020 | 2017 | 2. Finished recruiting, currently analysing |
| Brajcich, 2021 | 2017 | 1. Finished recruiting, manuscript submitted |
| Yohanna, 2021 | 2017 | 2. Finished recruiting, currently analysing |
| Apte, 2020 | 2018 | 3. Currently recruiting, including patients |
| Collins, 2020 | 2018 | 2. Finished recruiting, currently analysing |
| De Mik. 2020 | 2018 | 1. Finished recruiting, manuscript submitted |
| Mackay, 2020 | 2018 | 2. Finished recruiting, currently analysing |
| Schraa, 2020 | 2018 | 3. Currently recruiting, including patients |
| Smits, 2020 | 2018 | 1. Finished recruiting, manuscript submitted |
| Van der Sluijs, 2020 | 2018 | 2. Finished recruiting, currently analysing |
| Weller, 2020 | *?* | *Unknown* |
| Pagano, 2021 | 2019 | 2. Finished recruiting, currently analysing |
| Renz, 2021 | 2019? | *Unknown* |
| Douillet, 2021 | 2020 | 2. Finished recruiting, currently analysing |
| Malone, 2021 | 2020 | 3. Currently recruiting, including patients |
| Pourrat, 2021 | 2020 | 3. Currently recruiting, including patients |
| Raval, 2020 | 2020 | 3. Currently recruiting, including patients |

*Based on start date noted in trial register. **Evaluated between February and May 2022.
