# Supplementary material for: Alternative Randomized Trial Designs in Surgery: A Systematic Review
Source: Ann Surg. 2022 Jul 22;276(5):753–60. doi: 10.1097/SLA.0000000000005620 (PMC9534057; doi:10.1097/SLA.0000000000005620)
Supplement: SUPPLEMENTARY MATERIAL [file sla-276-0753-s005.docx]

**Supplement 5.**  Reported motivations for SW-RCTs

|  | **SW-RCTs** | | | | | | | | | | | | | | | | | | | | | | | | | **Total** | |
| --- | --- | --- | --- | --- | --- | --- | --- | --- | --- | --- | --- | --- | --- | --- | --- | --- | --- | --- | --- | --- | --- | --- | --- | --- | --- | --- | --- |
|  | Anderson* | Ayorinde* | Buhre^40^ | Deeken^41^ | De Mik* | Douillet* | Gilbert^42^ | Grossi | Lashoher^43^ | Mackay^53^ | Malone* | Noordman* | Pagano* | Peden^26^ | Pourrat | Raval | Schwarze^47^ | Sier^48^ | Smits* | Straatman* | Van der Sluijs* | Verberne^22^ | Weller* | Wilmink^50^ | Zatzick^52^ |  |  |
| Minimize contamination |  | X |  |  | X | X |  |  |  | X |  |  |  | X | X |  | X | X | X | X |  | X |  |  |  | 11 |  |
| All participants/centers are offered the intervention |  | X |  |  | X |  |  | X |  | X | X |  | X |  | X |  | X |  |  |  | X |  |  |  |  | 9 |  |
| Suitable in settings where it is impossible to randomize individual patients |  |  |  |  |  | X |  |  |  | X |  |  | X |  |  |  |  | X | X | X | X |  | X |  |  | 8 |  |
| Suitable for initiatives that are rolled out over time |  | X |  |  |  |  |  |  |  | X | X |  |  |  |  |  |  |  | X |  |  |  | X |  |  | 5 |  |
| Easier (national) implementation in clinical practice |  |  |  |  |  | X |  |  |  |  |  |  |  |  | X | X |  | X | X |  |  |  |  |  |  | 5 |  |
| Allowing analysis of the effect of time on intervention |  |  |  |  |  |  |  |  |  | X |  |  | X | X |  |  |  |  |  |  |  |  |  |  |  | 3 |  |
| Increased power |  | X |  |  |  |  |  |  |  | X |  |  |  | X |  |  |  |  | X |  |  |  |  |  |  | 4 |  |
| More efficient |  |  |  |  |  |  |  |  |  |  |  |  |  | X |  |  |  |  |  |  | X | X |  |  |  | 3 |  |
| Reduced risk of bias (due to within-cluster estimation of treatment effect) |  | X |  |  |  |  |  |  |  |  |  |  |  |  |  |  |  |  |  |  |  |  |  | X |  | 2 |  |
| Improves recruitment feasibility |  |  |  |  |  |  |  | X |  |  |  | X |  |  |  |  |  |  |  |  |  |  |  |  |  | 2 |  |
| Possibility to evaluate both effect of implementation and clinical outcomes |  |  |  |  |  |  |  |  |  |  | X |  |  |  |  | X |  |  |  |  |  |  |  |  |  | 2 |  |
| Reason specific for the trial itself |  |  |  |  | X |  |  | X |  |  |  |  |  |  |  |  |  |  |  |  |  |  |  |  |  | 2 |  |
| Increased external validity |  |  |  |  |  |  |  |  |  |  |  |  |  | X |  |  |  |  |  |  |  |  |  |  |  | 1 |  |
| Prevention of period effect |  |  |  |  |  |  | X |  |  |  |  |  |  |  |  |  |  |  |  |  |  |  |  |  |  | 1 |  |

SW-RCT: stepped wedge randomized controlled trial. *Reference of published protocols are depicted in Supplement 9.
