# Supplementary material for: Alternative Randomized Trial Designs in Surgery: A Systematic Review
Source: Ann Surg. 2022 Jul 22;276(5):753–60. doi: 10.1097/SLA.0000000000005620 (PMC9534057; doi:10.1097/SLA.0000000000005620)
Supplement: SUPPLEMENTARY MATERIAL [file sla-276-0753-s006.docx]

**Supplement 6.** Reported motivations for using a RB-RCTs and TwiCs

|  | **RB-RCTs** | | | | | | | | | | | | | | **SW + RB-RCTs** | | | | | | | | **TwiCs** | | | | | | | **Total** | |
| --- | --- | --- | --- | --- | --- | --- | --- | --- | --- | --- | --- | --- | --- | --- | --- | --- | --- | --- | --- | --- | --- | --- | --- | --- | --- | --- | --- | --- | --- | --- | --- |
|  | Apte* | Brajcich* | Collins* | Eslami* | Hedberg* | Petro^46^ | Renz* | Tastaldi^49^ | Møller^45^ | Woodle | Yohanna* | Masters^54^ | Lindholt^29^ | Ayordine^18^ | | Linder | Mackay^53^ | Malone* | Smits* | Verberne^22^ | Weller* | Couwenberg (2020)^21^ | | Couwenberg (2016)* | | Schraa* | | |  | |  |
| **Motivations for using a registry** | | | | | | | | | | | | | | | | | | | | | | | | | | | | | | | |
| Generalizability of the trial population / data | X |  | X |  | X |  | X | X |  | X |  | X |  |  | |  |  |  |  |  |  |  | | X | | X | | | 9 | |  |
| Efficient data collection  (less additional time/labor) | X | X | X |  | X |  | X |  |  |  |  |  |  | X | |  |  |  |  |  |  |  | |  | | X | | | 7 | |  |
| Optimize trial enrollment | X |  | X |  |  |  |  |  |  |  |  |  |  | X | |  | X |  |  |  |  |  | | X | | X | | | 6 | |  |
| Reduction of costs |  | X | X | X |  |  | X |  |  |  |  |  |  |  | |  | X |  |  |  |  |  | |  | | X | | | 6 | |  |
| High level of data quality |  |  |  |  | X |  |  |  | X |  |  |  |  |  | |  |  |  |  | X |  |  | |  | |  | | | 3 | |  |
| Quick implementation in other hospitals | X |  |  |  |  |  |  |  |  |  |  |  |  |  | |  |  |  |  |  |  |  | |  | | X | | | 2 | |  |
| Ascertainment of outcomes during trial and in follow-up |  |  |  |  |  |  |  |  |  | X | X |  |  |  | |  |  |  |  |  |  |  | |  | |  | | | 2 | |  |
| Facilitate long term follow up |  |  | X |  |  |  |  |  |  | X |  |  |  |  | |  |  |  |  |  |  |  | |  | |  | | | 2 | |  |
| Patient friendly |  |  |  |  |  |  |  |  |  |  |  |  |  | X | |  |  |  |  |  |  |  | |  | |  | | | 1 | |  |
| Ability to cross other with other countries registries |  |  |  |  | X |  |  |  |  |  |  |  |  |  | |  |  |  |  |  |  |  | |  | |  | | | 1 | |  |
| Possibility to include large groups of patients |  |  |  |  |  |  |  |  |  |  |  | X |  |  | |  |  |  |  |  |  |  | |  | |  | | | 1 | |  |
| Avoids prolonged recruitment (interim analysis can be planned easily to evaluate if goals are achieved) |  |  |  |  |  |  | X |  |  |  |  |  |  |  | |  |  |  |  |  |  |  | |  | |  | | | 1 | |  |
| Possibility to detect selection bias (comparing the trial population to all the patients in registry) |  |  |  |  |  |  | X |  |  |  |  |  |  |  | |  |  |  |  |  |  |  | |  | |  | | | 1 | |  |
| **Additional motivations for TwiCs** | | | | | | | | | | | | | | | | | | | | | | | | | | | | | | | |
| Avoid disappointment bias |  | | | | | | | | | | | | | | | | | | | | | | | | X | | X | 2 | | | |
| Overcome ethical dilemma’s |  |  |  |  |  |  |  |  |  |  |  |  |  |  |  |  |  |  |  |  |  |  |  |  |  | | X | 1 | | | |

RB-RCT: registry based randomized controlled trial. TwiCs: trials within cohorts. SW-RCT: stepped wedge randomized controlled trial. *Reference of published protocols are depicted in Supplement 9.
