# Supplementary material for: Alternative Randomized Trial Designs in Surgery: A Systematic Review
Source: Ann Surg. 2022 Jul 22;276(5):753–60. doi: 10.1097/SLA.0000000000005620 (PMC9534057; doi:10.1097/SLA.0000000000005620)
Supplement: SUPPLEMENTARY MATERIAL [file sla-276-0753-s007.docx]

**Supplement 7.**  Reported limitations for SW-RCTs

|  | **SW-RCTs** | | | | | | | | | | | | | | | | | | | | | | | | | |  | |
| --- | --- | --- | --- | --- | --- | --- | --- | --- | --- | --- | --- | --- | --- | --- | --- | --- | --- | --- | --- | --- | --- | --- | --- | --- | --- | --- | --- | --- |
|  | Anderson* | Ayorinde* | Buhre^40^ | Deeken^41^ | De Mik* | Douillet* | Gilbert^42^ | Grossi* | Lashoher^43^ | Mackay* | Malone* | Noordman* | Pagano* | Peden^26^ | Pourrat | Raval | Schwarze^47^ | Sier^48^ | Smits* | Straatman* | Van der Sluijs* | Verberne^22^ | Weller* | Wilmink^50^ | Zatzick^52^ | **Total** | |  |
| Confounding of treatment effect by time |  |  |  |  |  |  |  |  | X | X |  |  |  |  |  |  |  |  |  |  |  | X |  |  | X | 4 | |  |
| Risk of contamination |  |  |  |  |  |  |  |  |  | X |  |  |  |  |  |  |  |  | X |  |  |  |  |  |  | 2 | |  |
| Less efficient (in general) |  |  |  |  |  |  |  |  |  |  |  |  |  |  |  |  |  |  | X |  |  |  |  |  |  | 1 | |  |
| Enrollment challenges (no flexibility, study has to move forward) |  |  |  |  |  |  |  |  |  |  |  |  |  |  |  |  | X |  |  |  |  |  |  |  |  | 1 | |  |
| Overestimation of sample size ( fixed step lengths) |  |  |  |  |  |  |  |  |  |  |  |  |  |  |  |  |  |  |  |  | X |  |  |  |  | 1 | |  |
| Patients can participate in both protocols dependent on time of presentation |  |  |  |  |  |  |  |  |  |  |  |  |  |  |  |  |  |  |  |  |  | X |  |  |  | 1 | |  |
| Obstacle in sample size calculation |  |  |  |  |  |  |  |  |  |  |  |  |  |  |  |  |  |  |  |  |  |  | X |  |  | 1 | |  |
| Harmful when implemented strategy is not beneficial |  |  |  |  |  |  |  |  |  |  |  |  | X |  |  |  |  |  |  |  |  |  |  |  |  | 1 | |  |

SW-RCT: stepped wedge randomized controlled trial. *Reference of published protocols are depicted in Supplement 9.
