# Supplementary material for: Alternative Randomized Trial Designs in Surgery: A Systematic Review
Source: Ann Surg. 2022 Jul 22;276(5):753–60. doi: 10.1097/SLA.0000000000005620 (PMC9534057; doi:10.1097/SLA.0000000000005620)
Supplement: SUPPLEMENTARY MATERIAL [file sla-276-0753-s008.docx]

**Supplement 8.** Reported limitations for using a registry and TwiCs

|  | **RB-RCTs** | | | | | | | | | | | | | **SW + RB-RCTs** | | | | | | | | **TwiCs** | | | | **Total** |
| --- | --- | --- | --- | --- | --- | --- | --- | --- | --- | --- | --- | --- | --- | --- | --- | --- | --- | --- | --- | --- | --- | --- | --- | --- | --- | --- |
|  | Apte* | Brajcich* | Collins* | Eslami* | Hedberg* | Petro^46^ | Renz* | Tastaldi^49^ | Møller^45^ | Masters^44^ | Lindholt^29^ | Woodle^51^ | Yohanna* | Ayordine^18^ | LInder | Mackay* | Malone* | Smits* | Verberne^22^ | Weller* | | Couwenberg (2020)^21^ | Couwenberg (2016)* | | Schraa* |  |
| **Motivations for RB-RCTs** | | | | | | | | | | | | | | | | | | | | | | | | | | |
| Trial data is limited to variables included in the registry |  | X |  | X |  |  |  |  |  |  |  | X |  |  |  |  |  |  |  |  | |  |  | |  | 3 |
| Trial is limited to study population included in the registry |  | X |  |  |  |  | X |  |  |  |  |  | X |  |  |  |  |  |  |  | |  |  | |  | 3 |
| Validity of registry data can be uknown/poor |  | X |  |  |  |  |  |  |  |  |  |  | X |  |  |  |  |  |  |  | |  |  | |  | 2 |
| Adjustment sample size (loss to follow up) |  |  |  | X |  |  |  |  |  |  |  |  |  |  |  |  |  |  |  |  | |  |  | |  | 1 |
| Less controlled environment |  |  |  |  |  |  |  | X |  |  |  |  |  |  |  |  |  |  |  |  | |  |  | |  | 1 |
| Necessity of data use agreements |  | X |  |  |  |  |  |  |  |  |  |  |  |  |  |  |  |  |  |  | |  |  | |  | 1 |
| Timing of data processing can very among centers (problems with timing interim analysis) |  | X |  |  |  |  |  |  |  |  |  |  |  |  |  |  |  |  |  |  | |  |  | |  | 1 |
| **Additional limitations for TwiCs** | | | | | | | | | | | | | | | | | | | | | | | | | | |
| Selective refusal of intervention arm |  | | | | | | | | | | | | | | | | | | | | X | | |  |  | 1 |
| Risk of over stratification (multiple trials) |  |  |  |  |  |  |  |  |  |  |  |  |  |  |  |  |  |  |  |  |  | | | X |  | 1 |
| Blinding is impossible |  |  |  |  |  |  |  |  |  |  |  |  |  |  |  |  |  |  |  |  |  | | | X |  | 1 |
| Internal validity bias |  |  |  |  |  |  |  |  |  |  |  |  |  |  |  |  |  |  |  |  |  | | |  | X | 1 |
| Potential low statistical power |  | | | | | | | | | | | | | | | | | | | |  | | |  | X | 1 |

RB-RCT: registry based randomized controlled trial. TwiCs: trials within cohorts. SW-RCT: stepped wedge randomized controlled trial. *Reference of published protocols are depicted in Supplement 9.
