# Supplementary material for: Alternative Randomized Trial Designs in Surgery: A Systematic Review
Source: Ann Surg. 2022 Jul 22;276(5):753–60. doi: 10.1097/SLA.0000000000005620 (PMC9534057; doi:10.1097/SLA.0000000000005620)
Supplement: SUPPLEMENTARY MATERIAL [file sla-276-0753-s009.docx]

**Supplement 9.** References of published protocols

| **SW-RCTs** | **Reference** |
| --- | --- |
| Anderson, 2020 | Anderson DJ, Ilieş I, Foy K, et al. Early recognition and response to increases in surgical site infections using optimized statistical process control charts—the Early 2RIS Trial: a multicenter cluster randomized controlled trial with stepped wedge design. Trials. 2020;21(1):1-10. doi:10.1186/s13063-020-04802-4 |
| Ayorinde, 2019 | Ayorinde JO, Summers DM, Pankhurst L, et al. PreImplantation Trial of Histopathology In renal Allografts (PITHIA): a stepped-wedge cluster randomised controlled trial protocol. BMJ Open. 2019;9(1):e026166. doi:10.1136/bmjopen-2018-026166 |
| De Mik, 2020 | de Mik SML, Stubenrouch FE, Legemate DA, Balm R, Ubbink DT. Improving shared decision-making in vascular surgery by implementing decision support tools: study protocol for the stepped-wedge cluster-randomised OVIDIUS trial. BMC Med Inform Decis Mak. 2020;20(1):172. doi:10.1186/s12911-020-01186-y |
| Douillet, 2021 | Douillet D, Riou J, Thoma M, et al. Thromboembolic risk stratification by TRiP(cast) score to rationalise thromboprophylaxis in patients with lower leg trauma requiring immobilisation: A study protocol of the casting stepped-wedge cluster randomised trial. BMJ Open. 2021;11(6):1-8. doi:10.1136/bmjopen-2020-04590 |
| Grossi, 2018 | Grossi U, Stevens N, McAlees E, et al. Stepped-wedge randomised trial of laparoscopic ventral mesh rectopexy in adults with chronic constipation: study protocol for a randomized controlled trial. Trials. 2018;19(1):90. doi:10.1186/s13063-018-24 |
| Mackay, 2020 | MacKay TM, Smits FJ, Latenstein AEJ, et al. Impact of nationwide enhanced implementation of best practices in pancreatic cancer care (PACAP-1): A multicenter stepped-wedge cluster randomized controlled trial. Trials. 2020;21(1):1-18. doi:10.1186/s13063-020-4180-z |
| Malone, 2021 | Malone S, McKay VR, Krucylak C, et al. A cluster randomized stepped-wedge trial to de-implement unnecessary post-operative antibiotics in children: the optimizing perioperative antibiotic in children (OPerAtiC) trial. Implement Sci. 2021;16(1):1-11. doi:10.1186/s13012-021-01096-1 |
| Noordman, 2018 | Noordman BJ, Wijnhoven BPL, Lagarde SM, et al. Neoadjuvant chemoradiotherapy plus surgery versus active surveillance for oesophageal cancer: A stepped-wedge cluster randomised trial. BMC Cancer. 2018;18(1):1-12. doi:10.1186/s12885-018-4034-1 |
| Pagano, 2021 | Pagano E, Pellegrino L, Rinaldi F, et al. Implementation of the ERAS (Enhanced Recovery after Surgery) protocol for colorectal cancer surgery in the Piemonte Region with an Audit and Feedback approach: Study protocol for a stepped wedge cluster randomised trial: A study of the EASY-NET project. BMJ Open. 2021;11(6):1-6. doi:10.1136/bmjopen-2020-047491 |
| Pourrat, 2021 | Pourrat X, Berthy E, Dupuis A, et al. Implementing a personalized pharmaceutical plan in kidney or liver transplant patients: study protocol for a stepped-wedge cluster randomized trial (GRePH). Trials. 2021;22(1):1-9. doi:10.1186/s13063-021-05749-w |
| Raval, 2020 | Raval M V., Wymore E, Ingram MCE, Tian Y, Johnson JK, Holl JL. Assessing effectiveness and implementation of a perioperative enhanced recovery protocol for children undergoing surgery: study protocol for a prospective, stepped-wedge, cluster, randomized, controlled clinical trial. Trials. 2020;21(1):1-13. doi:10.1186/s13063-020-04851- |
| Smits, 2020 | Smits FJ, Henry AC, Van Eijck CH, et al. Care after pancreatic resection according to an algorithm for early detection and minimally invasive management of pancreatic fistula versus current practice (PORSCH-trial): Design and rationale of a nationwide stepped-wedge cluster-randomized trial. Trials. 2020;21(1):1-16. doi:10.1186/s13063-020-4167-9 |
| Straatman, 2015 | Straatman J, Cuesta MA, Schreurs WHH, et al. The PRECious trial PREdiction of Complications, a step-up approach, CRP first followed by CT-scan imaging to ensure quality control after major abdominal surgery: Study protocol for a stepped-wedge trial. Trials. 2015;16(1):1-8. doi:10.1186/s13063-015-0903-y |
| Van der Sluijs, 2020 | van der Sluijs R, Fiddelers AAA, Waalwijk JF, et al. The impact of the Trauma Triage App on pre-hospital trauma triage: design and protocol of the stepped-wedge, cluster-randomized TESLA trial. Diagnostic Progn Res. 2020;4(1):1-8. doi:10.1186/s41512-020-00076-1 |
| Weller, 2020 | Weller J, Long JA, Beaver P, et al. Evaluation of the effect of multidisciplinary simulation-based team training on patients, staff and organisations: Protocol for a stepped-wedge cluster-mixed methods study of a national, insurer-funded initiative for surgical teams in New Zealand public hospitals. BMJ Open. 2020;10(2):1-8. doi:10.1136/bmjopen-2019-032997 |

SW-RCTs = stepped-wedge randomized controlled trials.

| **RB-RCTs** | **Reference** |
| --- | --- |
| Apte, 2020 | Apte SS, Moloo H, Jeong A, et al. Prospective randomised controlled trial using the REthinking Clinical Trials (REaCT) platform and National Surgical Quality Improvement Program (NSQIP) to compare no preparation versus preoperative oral antibiotics alone for surgical site infection rates in elective colon surgery: a protocol. BMJ Open. 2020;10(7):e036866. doi:10.1136/bmjopen-2020-03686 |
| Brajcich, 2021 | Brajcich BC, Ko CY, Liu JB, Ellis RJ, D′Angelica MI. A NSQIP-based randomized clinical trial evaluating choice of prophylactic antibiotics for pancreaticoduodenectomy. J Surg Oncol. 2021;123(6):1387-1394. doi:10.1002/jso.26402 |
| Collins, 2020 | Collins MG, Fahim MA, Pascoe EM, et al. Study Protocol for Better Evidence for Selecting Transplant Fluids (BEST-Fluids): A pragmatic, registry-based, multi-center, double-blind, randomized controlled trial evaluating the effect of intravenous fluid therapy with Plasma-Lyte 148 versus 0.9% saline on delayed graft function in deceased donor kidney transplantation. Trials. 2020;21(1):1-19. doi:10.1186/s13063-020-04359-2 |
| Eslami, 2015 | Eslami MH, Doros G, Goodney PP, et al. Using vascular quality initiative as a platform for organizing multicenter, prospective, randomized clinical trials: Overpar trial. Ann Vasc Surg. 2015;29(2):278-285. doi:10.1016/j.avsg.2014.08.007 |
| Hedberg, 2019 | Hedberg S, Olbers T, Peltonen M, et al. BEST: Bypass equipoise sleeve trial; rationale and design of a randomized, registry-based, multicenter trial comparing Roux-en-Y gastric bypass with sleeve gastrectomy. Contemp Clin Trials. 2019;84(July):105809. doi:10.1016/j.cct.2019.07.001 |
| Renz, 2021 | Renz BW, Adrion C, Klinger C, et al. Pylorus resection versus pylorus preservation in pancreatoduodenectomy (PyloResPres): Study protocol and statistical analysis plan for a German multicentre, single-blind, surgical, registry-based randomised controlled trial. BMJ Open. 2021;11(11). doi:10.1136/bmjopen-2021-056191 |
| Yohanna, 2021 | Yohanna S, Naylor KL, Mucsi I, et al. A Quality Improvement Intervention to Enhance Access to Kidney Transplantation and Living Kidney Donation (EnAKT LKD) in Patients With Chronic Kidney Disease: Clinical Research Protocol of a Cluster-Randomized Clinical Trial. Can J Kidney Heal Dis. 2021;8. doi:10.1177/2054358121997266 |

RB-RCTs = registry based randomized controlled trials.

| **TwiCs** | **Reference** |
| --- | --- |
| Couwenberg, 2016^55^ | Couwenberg AM, Burbach MJP, Smits AB, et al. The impact of retractor SPONGE-assisted laparoscopic surgery on duration of hospital stay and postoperative complications in patients with colorectal cancer (SPONGE trial): Study protocol for a randomized controlled trial. Trials. 2016;17(1):1-7. doi:10.1186/s13063-016-1256-x |
| Schraa, 2020^32^ | Schraa SJ, Van Rooijen KL, Van Der Kruijssen DEW, et al. Circulating tumor DNA guided adjuvant chemotherapy in stage II colon cancer (MEDOCC-CrEATE): Study protocol for a trial within a cohort study. BMC Cancer. 2020;20(1):1-10. doi:10.1186/s12885-020-07252-y |

TwiCs = trials within cohorts.
